# Gastrodin plays a protective role in alleviating hepatic ischemia reperfusion injury by regulating heme oxygenase-1 expression

**DOI:** 10.1590/1414-431X2024e14248

**Published:** 2025-02-03

**Authors:** Shan-Shan He, Han-Fei Huang, Shao-Qing Shi, Jing-Jiao Zhao, Bo Yuan, Xiang Ji, Hong-Bin Zhang

**Affiliations:** 1 The First Affiliated Hospital of Kunming Medical University, Kunming, Yunnan, China

**Keywords:** Anti-apoptotic factors, Gastrodin, HO-1, Hepatic ischemia reperfusion injury, Inflammatory factors, Oxidative stress factors

## Abstract

Hepatic ischemia reperfusion injury (HIRI) is a pathophysiological and complex systemic process involving multiple tissues and organs. Gastrodin (GSTD), a natural compound from *Gastrodia elata*, displays a variety of interesting pharmacological activities. Heme oxygenase-1 (HO-1), a stress-responsive protein, has a cytoprotective defense response against oxidative and inflammatory injuries. The aim of this investigation was to elucidate whether GSTD plays a protective role against HIRI by regulating HO-1 expression. GSTD (100 mg/kg) or zinc protoporphyrin (15 mg/kg; an HO-1 inhibitor) was administered to HIRI C57 male mice. GSTD decreased glutamic pyruvic transaminase and glutamic oxaloacetic transaminase levels in HIRI mice. Inflammatory (TNF-α and IL-6) and oxidative-stress (malondialdehyde, MDA) markers of HIRI mice were decreased by GSTD. GSTD up-regulated HO-1 protein and mRNA expression in HIRI mice but decreased caspase-3 and -9 protein expression. GSTD lowered mRNA expression of apoptosis-related genes (caspase-3, -9, -12, and *Bax*) in the liver of HIRI mice but enhanced mRNA level of the anti-apoptotic *Bcl*-2 gene. Consistent with *in vivo* results, GSTD displayed a similar regulatory effect on the expression of mRNA (*HO*-1, caspase-3, -9, -12, *Bax*, and *Bcl*-2) and protein (HO-1, caspase-3 and -9) as well as inflammatory (TNF-α and IL-6) and on oxidative stress factors (superoxide dismutase and MDA) in BRL-3A cells transfected with small interfering HO-1 RNA in a hypoxia-reperfusion model. In conclusion, GSTD up-regulated HO-1 expression to play a protective role in HIRI by anti-apoptotic, anti-inflammatory, and antioxidant effects. GSTD is a promising natural compound that alleviated HIRI in liver surgery.

## Introduction

Hepatic ischemia reperfusion injury (HIRI) refers to the phenomenon in which the liver is injured after transient ischemia and hypoxia. The structural and functional damage to the liver is not relieved but aggravated after reperfusion is restored (1). HIRI occurs mainly during liver transplantation, partial hepatectomy, hypovolemic shock, and trauma (2). Marginal livers during transplantation are highly sensitive to HIRI stress and frequently show early allograft dysfunction, primary non-function, and further rejection (3). HIRI is not only one of the main causes of abnormal liver function after partial hepatectomy, donor liver dysfunction, and liver failure after liver transplantation, but is also one of the main bottlenecks in the development of liver surgery. HIRI occurs successively in two stages. In the ischemic stage, hepatocyte apoptosis increases significantly due to massive glycogen consumption, a weakened oxygen supply, and decreased ATP production in the liver tissue. In the reperfusion stage, metabolic disorders occur in the liver tissue, resulting in the activation of the inflammatory cascade and the release of various inflammatory and oxidative stress factors (4). Currently, several strategies exist to alleviate the effect of HIRI (5-8), including minimizing the time for liver ischemia, improving ischemic tissue metabolism, scavenging free radicals, reducing calcium overload, and ischemic preconditioning. However, no means exist to completely avoid the harmful effects of HIRI. The discovery of new drugs and methods to lessen the negative effect of HIRI is still necessary.

*Gastrodia elata* is a common herbal medicine that has been used for more than two thousand years in China. Gastrodin (GSTD; 4-hydroxymethylbenzol-β-D-glucopyranoside hemihydrate) is the well-known active component responsible for the pharmacological action of *Gastrodia elata*. GSTD was demonstrated to reduce peripheral vascular resistance, increase cardiovascular and cerebrovascular blood flow, improve circulation, have analgesic, sedative, and hypnotic effects, and enhance immunity (9-12). GSTD-containing preparations are extensively used in the treatment of cardiovascular and cerebrovascular diseases in China (13). Several studies have revealed that GSTD is effective in protecting spinal cord ischemia reperfusion injury and cerebral ischemia reperfusion injury (14,15). Our previous study demonstrated that GSTD alleviated H_2_O_2_-induced oxidative stress in liver sinusoidal endothelial cells in mice through p38 MAPK phosphorylation (16).

Heme oxygenase-1 (HO-1) is a rate-limiting enzyme in heme catabolism, and generates biliverdin, free iron, and carbon monoxide (17). It has been shown that HO-1 is a stress-responsive protein that has a cytoprotective defense response against oxidative injury. HO-1 overexpression exerts potent adaptive anti-inflammatory and anti-apoptotic effects in several transplantation models (18,19). HO-1 induction was cytoprotective in rat liver transplant and extended-organ cold ischemia models (20). In a previous study, we reported how *in vivo* experiments demonstrated that the HO-1 mRNA level was up-regulated by GSTD in a HIRI mouse model, suggesting that GSTD may protect against HIRI injury by regulating HO-1 (21).

In this study, GSTD was administered to a HIRI mouse model in an *in vivo* experiment. Small interfering HO-1 cells were constructed and treated with GSTD in *in vitro* experiments.

## Material and Methods

### Animal experiments

C57 male mice weighing 20-30 g were grouped into sham, hepatic ischemia-reperfusion injury (HIRI), HIRI+GSTD, HIRI+GSTD+zinc protoporphyrin (ZnPP), and HIRI+ZnPP groups, with eight mice in each group. ZnPP was used as an inhibitor of HO-1. Prior to the establishment of the HIRI model, the mice were given an equal volume of saline every day for 3 days. The abdominal cavity of anesthetized mice (10% chloral hydrate) was opened using surgical scissors. The blood vessels of the middle hepatic and left hepatic lobes were clamped using a vascular clamp. Another large vascular clamp was used to clamp the skin in the surgical wound to temporarily close the abdomen. After 1 h of ischemia, the abdomen was opened again and the blood vessels were loosened for 30 s. Then, the abdominal cavity was closed by surgical suture. The mice were provided with standard food and water after waking. Following 6 h of perfusion, the mice were euthanized. Liver tissues and blood samples from the inferior vena cava, middle hepatic lobe, and left hepatic lobe were collected. The sham group did not undergo vascular clamping and perfusion but were anesthetized and the abdominal cavity opened as with the HIRI group. Drugs were given 3 days before the model was established, with 100 mg/kg GSTD or 15 mg/kg ZnPP each day by intraperitoneal injection. The study was approved by the Ethics Committee of Yunnan Medical University, China (Ethics No. kmmu20230526).

### Liver function test

Blood samples were stored at -20°C or -80°C, thawed, and centrifuged (1006.2 *g*, 15 min, 2-8°C) prior to each test. Heparin was used as an anticoagulant for plasma. Then, levels of glutamic pyruvic transaminase (ALT) and glutamic oxaloacetic transaminase (AST) in the samples were examined via an automatic biochemical analyzer (Rayto Life and Analytical Sciences Co., Ltd., China).

### Hematoxylin-eosin staining

Paraffin sections of the liver were incubated for 1 h at 64°C. Subsequently, they were de-waxed and stained with hematoxylin-eosin (H&E) or rehydrated by washing in 100% xylene, 100% ethanol, 95% ethanol, 80% ethanol, 70% ethanol, and afterwards washed in phosphate-buffered saline (Solarbio, China).

### Detection of apoptosis

Terminal deoxynucleotidyl transferase dUTP nick end labeling (TUNEL) apoptosis detection kits (Beyotime Biotech Inc., China) were used to monitor nuclear DNA fragmentation in the late apoptotic process of cells. A 4′,6-diamidino-2-phenylindole (DAPI) solution was used to dye nuclei. Finally, the samples were analyzed under a fluorescence microscope (Nikon, Japan). Apoptotic and non-apoptotic nuclei were stained blue by DAPI, and red fluorescence localized by TMR-5-dUTP incorporation was found only in apoptotic nuclei.

### Western blotting

Tissue samples were lysed with radioimmunoprecipitation buffer (Servicebio, China). Tissue homogenate was centrifuged at 16000 *g* for 15 min at 4°C. After that, the protein concentration was calculated using a BCA protein quantification kit (Beyotime Biotechnology, China). Proteins in samples were separated on a 10% SDS-polyacrylamide electrophoresis gel and then transferred onto polyvinylidene fluoride (PVDF) membranes (Millipore, USA) at 4°C, 300 mA for 1 h. The PVDF membranes were rinsed with Tris buffered saline with 5% Tween (TBST) once, then blocked in 5% BSA for 30 min at 37°C. In turn, the PVDF membranes were subjected to overnight incubation at 4°C with primary antibody and then at room temperature for 1 h with secondary antibody. Exposure was achieved on a gel imaging system with ECL chemiluminescence substrate (Biosharp, China).

### Quantitative PCR

TRIzol reagent (Ambion, USA) was used to isolate total RNA, which was quantified using a Nanodrop spectrophotometer (Thermo Fisher Scientific Inc., USA). Copy DNA was generated using a SureScript-First-strand-cDNA-synthesis-kit (Servicebio). The quantitative PCR (qPCR) reaction system was made up of cDNA (3 μL), BlazeTaq qPCR Mix (5 μL), and upstream and downstream primer (1 μL, Table 1) The reactions were performed on a CFX96 real-time quantitative fluorescence PCR instrument (Bio-Rad, USA). The amplification reaction was programmed as a 1-min pre-denaturation at 95°C, preceded by 40 cycles, each consisting of 20 s at 95°C, 20 s at 55°C, and 30 s at 72°C. *β*-actin was used as a reference. The relative expression of mRNA was calculated according to the 2-ΔΔCt method.

**Table 1:** Primers of genes used in the q-PCR assay.

Primer name	Sequence (5′-3′)
GAPDH	F: CCTTCCGTGTTCCTACCCC
GAPDH	R: GCCCAAGATGCCCTTCAGT
Bax	F: ACAGATCATGAAGACAGGGG
Bax	R: AAAGTAGAAGAGGGCAACCA
Bcl-2	F: CTGGCATCTTCTCCTTCC
Bcl-2	R: GAGTTCCTCCACCACCGT
Caspase-3	F: AGGAAGATGGGGAGAGCG
Caspase-3	R: CCTGAGGGTGGGGTGAGA
Caspase-9	F: GGGACTCACAGCAAAGGA
Caspase-9	R: GATGACCACCACAAAGCA
HO-1	F: CACATCCAAGCCGAGAAT
HO-1	R: CGGGAAGGTAAAAAAAGC
Caspase-12	F: AAAGACAGAAAGGGCAAAA
Caspase-12	R: TCAGACTCCGACAGTTAGA

### Enzyme-linked immunosorbent assay

The levels of tumor necrosis factor (TNF)-α, superoxide dismutase (SOD), interleukin (IL)-6, and malondialdehyde (MDA) in liver tissues were determined by enzyme-linked immunosorbent assay (ELISA). MDA and SOD ELISA kits were purchased from NanJing JianCheng (China). The IL-6 kits were purchased from Neobioscience Technology Company (China).

### Cell culture and small interfering RNA transfection

BRL-3A cells were thawed and cultured at 37°C in a 5% CO_2_ incubator, then transferred into T-25 culture flasks. The cells were passaged at 1:3 when they covered 80% of the flasks. Small interfering (si) RNAs (si-HO-1-001, si-HO-1-002, si-HO-1-003, and si-NC (negative control)) were made by Changsha Abiwell Biotechnology Co., Ltd. (China). Cells were incubated with transfection complex for 6 h and the medium was changed to normal medium. After 48 h, the cells were collected for further analysis.

### Construction of HIRI cell model

The cell model groups used in this study included normal control (N group), hypoxia-reperfusion (H/R group), G1 (H/R+10 µM GSTD), G2 (H/R+50 µM GSTD group), G3 (H/R+100 µM GSTD group), normal saline (NS group), and a G3+si-HO-1 group. BRL-3A or si-HO-1 BRL-3A cells (1×10^6^) in the logarithmic growth phase were seeded onto 6-well plates containing DMEM medium and sealed in a wooden box with a hole in two opposing sides. The box was placed in an incubator and two injection hoses were respectively connected to a hole on two sides of the box. A mixture of 94% N_2_, 5% CO_2_, and 1% O_2_ was supplied from one end of the hose. The other end of the hose was connected to an oxygen analyzer to monitor the oxygen content. When the oxygen content was a steady 1% in the box, the two ends of the injection hose were clamped to establish a hypoxic atmosphere. Cells were cultured in the box for 6 h. GSTD was added to cells and these cells were cultured in DMEM for another 1 h in 95% O_2_ and 5% CO_2_. The joints and lid of the box were waxed to avoid leakage.

### Molecular docking

The structure of HO-1 was utilized as a receptor. The two-dimensional chemical structure of GSTD was downloaded from the PubChem database. By optimizing Autodock 4.21 software, GSTD interacted with HO-1 at a molecular stimulating level. The docking center was set as X 1/4 16.97, Y 1/4 59.62, and Z 1/4 1.99, and the number of points was set as X 1/4 25, Y 1/4 25, and Z 1/4 25, individually (19). The spacing was modified in 0.3750 and the other parameters were the default.

### Statistical analysis

Statistical analysis was performed using one-way analysis of variance, and was based on at least three different experiments. The results were considered to be statistically significant when P<0.05. All statistical tests were carried out using the computer program SPSS (SPSS Inc., IBM, USA).

## Results

### GSTD alleviated HIRI in a mouse model

**Figure 1:**
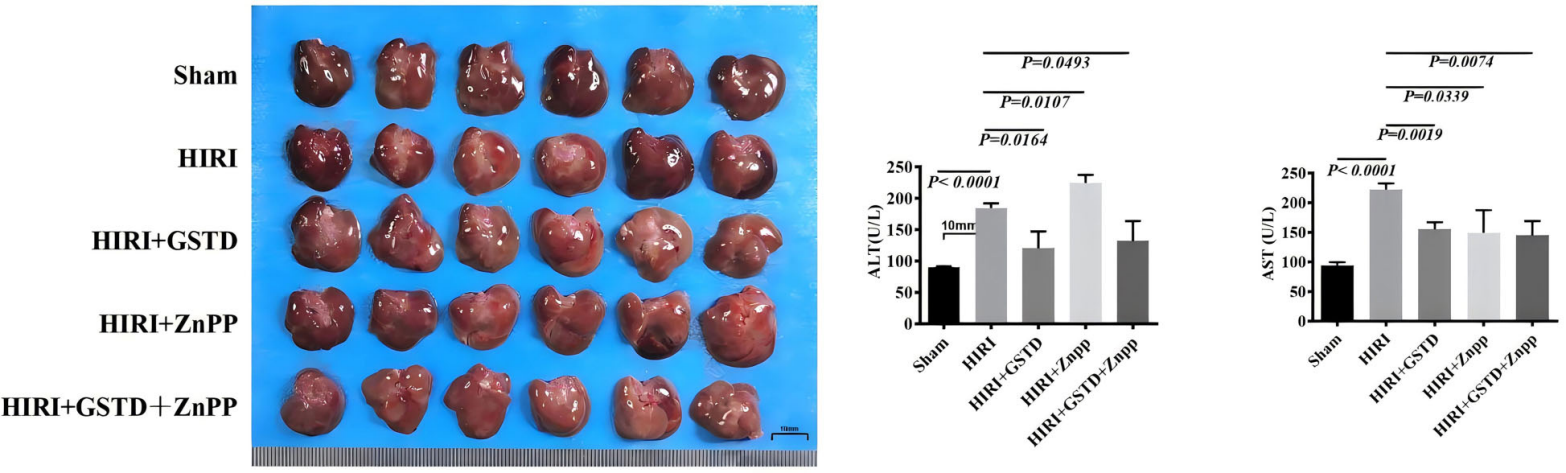
Effect of gastrodin (GSTD) on the liver tissue (left panel, scale bar 10 mm), glutamic pyruvic transaminase (ALT), and glutamic oxaloacetic transaminase (AST) (right panel) in hepatic ischemia reperfusion injury (HIRI) mice. Data are reported as means and SD (ANOVA). ZnPP: zinc protoporphyrin (HO-1 inhibitor).

**Figure 2:**
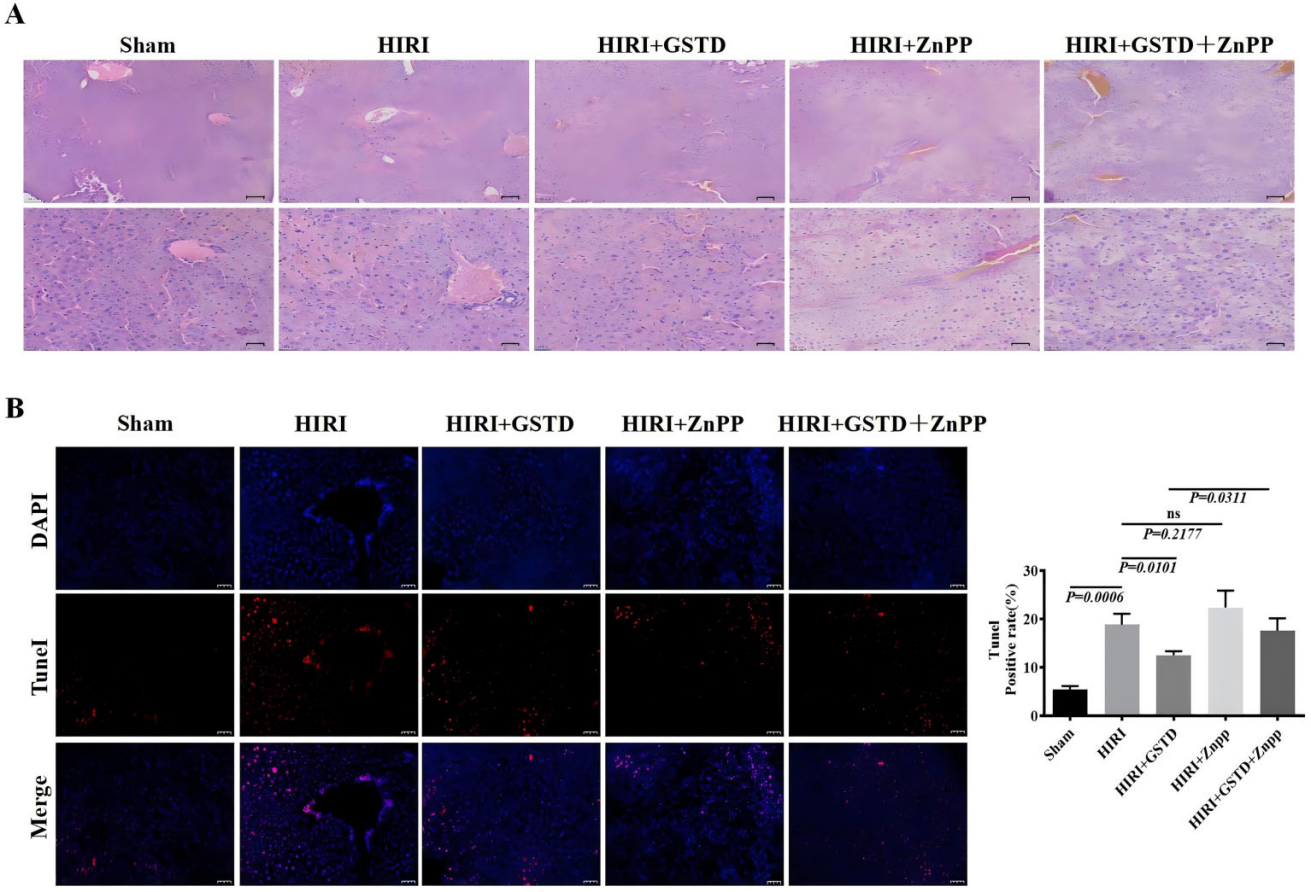
Effect of gastrodin (GSTD) on liver tissue morphology and cell apoptosis in hepatic ischemia reperfusion injury (HIRI) mice. **A**, Liver tissue morphology. **B**, TUNEL staining and histogram of cell apoptosis. Data are reported as means and SD (ANOVA). ZnPP: zinc protoporphyrin (HO-1 inhibitor). Scale bars: 50 μm.

The livers of mice in the sham group were relatively intact; those from mice in the other groups showed varying degrees of damage with the most serious damage in the HIRI model group (Figure 1, left panel). The levels of ALT and glutamic AST in the liver of mice in the HIRI group were notably higher than those of the sham group. Liver ALT and glutamic AST of mice in the HIRI+GSTD group were significantly lower than those in the HIRI group (Figure 1, right panel). These observations suggested that GSTD can reduce levels of ALT and glutamic AST in HIRI mice. In addition, liver levels of ALT and AST of mice of the HIRI+ZnPP group were significantly higher than those in the HIRI group, demonstrating that ZnPP had a negative effect on HIRI. Gastrodin, however, reversed the negative effect of ZnPP in HIRI, which was demonstrated by a significant reduction of ALT and AST liver levels of HIRI+GSTD+ZnPP mice compared to those in the HIRI+ZnPP group (Figure 1, right panel). Liver tissues of mice in the HIRI group displayed a higher level of swelling/necrosis, steatosis, and inflammatory cell infiltration than those of mice in the sham group (Figure 2A). Mice in the GSTD+HIRI group displayed a reduced level of tissue swelling/necrosis and inflammatory cell infiltration than those in the HIRI group. GSTD alleviated liver damage due to HIRI. The worse liver damage due to ZnPP in HIRI mice could also be slightly reversed by GSTD, indicated by a reduced level of liver swelling/necrosis and inflammatory cell infiltration in mice of the HIRI+GSTD+ZnPP group compared to those of the HIRI+ZnPP group (Figure 2A). Findings from TUNEL staining demonstrated that the liver apoptosis of mice in the HIRI group was notably greater than that of mice in the sham group (Figure 2B). ZnPPcan further induced liver apoptosis as shown by an elevated level of TUNEL staining in mice of the HIRI+ZnPP compared to HIRI group. However, the liver apoptosis noted in the HIRI mice was reduced by GSTD since TUNEL staining in mice of the HIRI+GSTD group was weaker than in mice of the HIRI group. ZnPP-induced liver apoptosis was also relieved by GSTD treatment as demonstrated by a significant decrease in TUNEL staining in mice of the HIRI+GSTD+ZnPP group relative to those in the HIRI+ZnPP group (Figure 2B).

### GSTD decreased liver apoptosis of HIRI mice by targeting HO-1 expression

**Figure 3:**
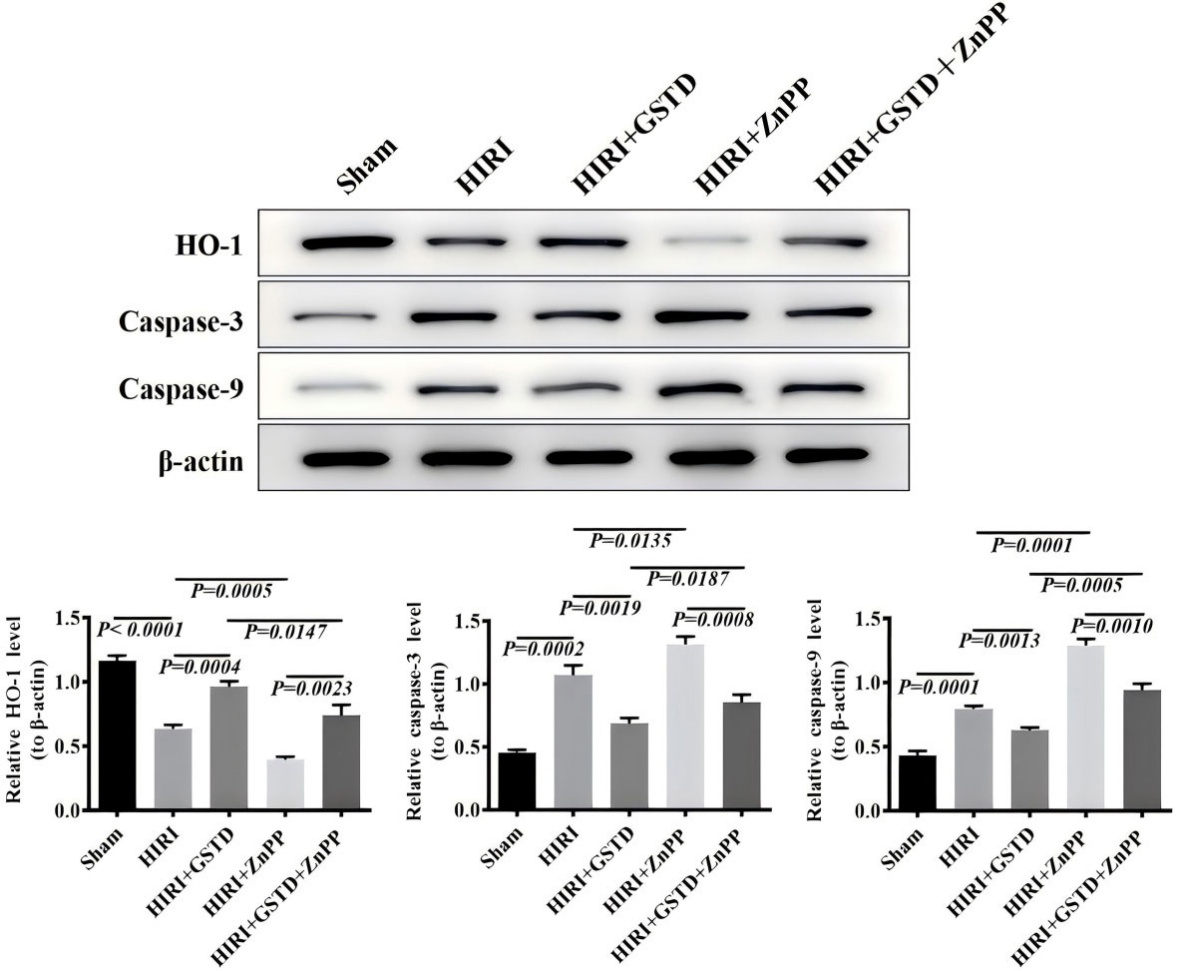
Effect of gastrodin (GSTD) on heme oxygenase-1 (HO-1), caspase-3, and caspase-9 protein expression in liver tissues of hepatic ischemia reperfusion injury (HIRI) mice. Data are reported as means and SD (ANOVA). ZnPP: zinc protoporphyrin.

Caspase-3 and -9 are two typical genes associated with apoptosis in the mitochondrial pathway (22,23). As shown in Figure 3, the expression levels of caspase-3 and -9 proteins in the liver of mice in the HIRI group were significantly higher than those of mice in the sham group. Caspase-3 and -9 were activated and responsible for liver apoptosis in HIRI. However, caspase-3 and -9 protein levels in the liver of mice of the HIRI+GSTD group were significantly lower than those in the HIRI group, indicating the anti-apoptotic effect of GSTD in the HIRI model. ZnPP exerted an inductive effect on the expression of caspase-3 and -9 proteins as indicated by a higher level of caspase-3 and -9 proteins in the liver of mice in the ZnPP+HIRI group compared to those in the HIRI group. A decrease in caspase-3 and -9 protein levels by GSTD was also concluded from a decrease in the liver expression of the two proteins in the ZnPP+HIRI+GSTD group compared to those in the ZnPP+HIRI group. HO-1 liver expression of mice of the HIRI group was significantly down-regulated compared with that in the sham group. Mice in the GSTD+HIRI group displayed a higher liver level of HO-1 protein expression than those in the HIRI group, suggesting an antagonistic role of GSTD on the down-regulation of HO-1 in HIRI mice. HO-1 liver expression of mice in the ZnPP+HIRI group was significantly lower than in the HIRI group. However, mice in the ZnPP+HIRI+GSDT group showed a higher liver level of HO-1 protein expression than those of mice in the ZnPP+HIRI group. Taken together, it was confirmed that GSTD can up-regulate HO-1 expression in HIRI mice.

**Figure 4:**
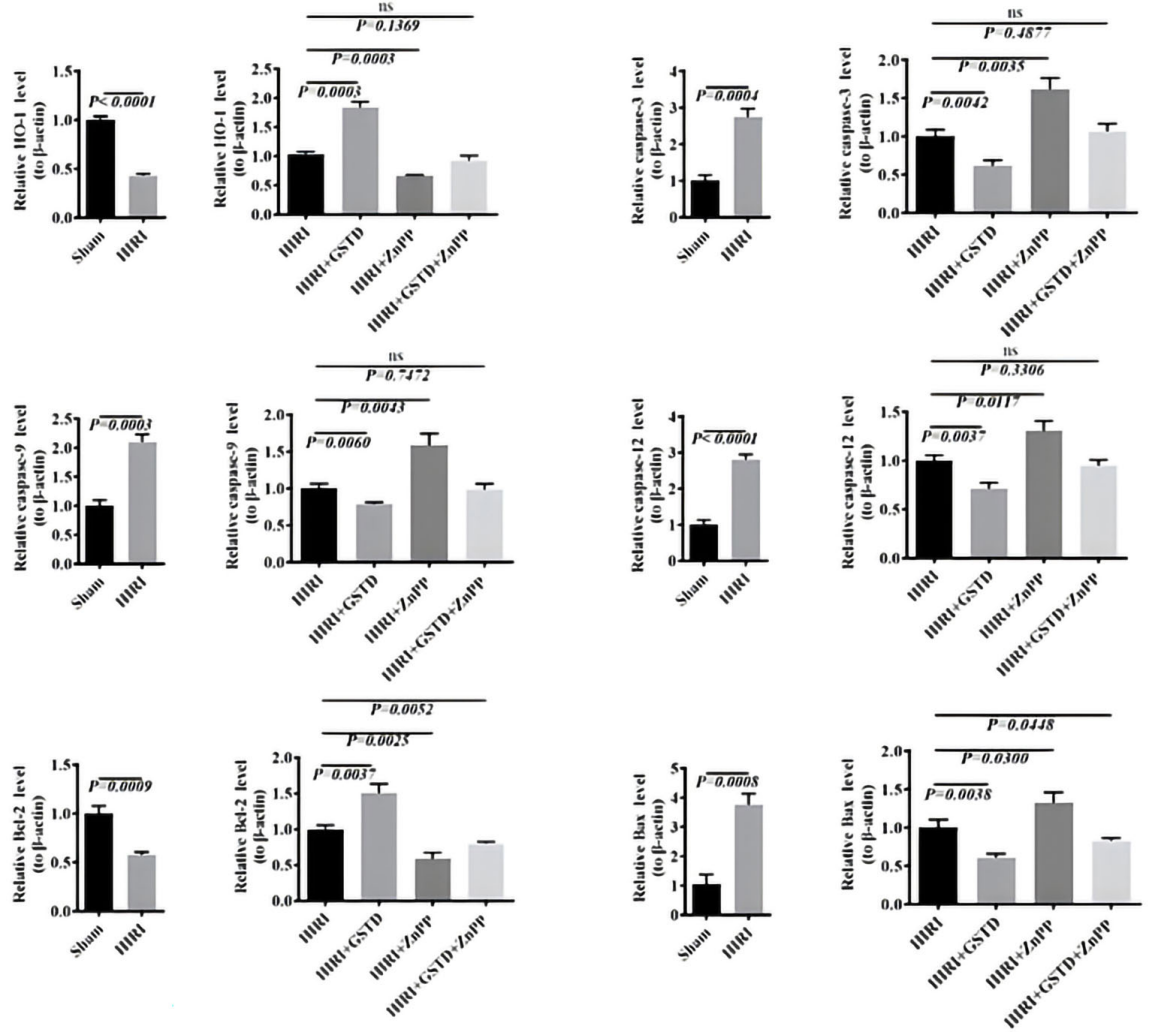
Effect of gastrodin (GSTD) on m-RNA level expression of caspase-3, -9, -12, *Bcl-2*, and *Bax* in liver tissues of hepatic ischemia reperfusion injury (HIRI) mice. Data are reported as means and SD (ANOVA; ns, not significant). HO-1: heme oxygenase-1; ZnPP: zinc protoporphyrin (HO-1 inhibitor).

To verify results, qPCR was employed to measure mRNA expression levels of *HO-1*, caspase-3, -9, -12, *Bcl-2*, and *Bax* in liver tissues (Figure 4). The RNA liver levels of apoptosis-related genes, including caspase-3, -9, -12, and *Bax*, were consistently up-regulated in mice in the HIRI group compared to those in the sham group. GSTD can suppress the mRNA up-regulation of such apoptosis-related genes since the mice in the GSTD+HIRI group showed higher liver mRNA levels of caspase-3, -9, -12, and *Bax* than in the HIRI group. Mice in the ZnPP+HIRI group displayed higher liver mRNA expression of caspase-3, -9, -12, and *Bax* than mice of the HIRI group. However, mice in the ZnPP+HIRI+GSTD group showed lower liver mRNA levels of caspase-3, -9, -12, and *Bax* than those in the ZnPP+HIRI group. Although ZnPP did up-regulate RNA levels of apoptosis-related genes in HIRI mice, GSTD repressed the up-regulatory effect due to ZnPP. However, the liver mRNA level of anti-apoptosis *Bcl-2* was significantly down-regulated in mice in the HIRI group relative to those in the Sham group. The liver *Bcl-2* mRNA level of mice of the GSTD+HIRI group was significantly higher than in the HIRI group, illustrating that *Bcl-2* down-regulation due to HIRI was partially reversed by GSTD. Mice in the ZnPP+GSTD+HIRI group showed a higher liver mRNA level of *Bcl-2* than those in the ZnPP+HIRI group. This also demonstrated that GSTD suppressed the mRNA down-regulation of *Bcl-2* in HIRI mice. The *HO-1* mRNA expression model was quite similar to that of *Bcl-2*. Although the *HO-1* mRNA level was significantly down-regulated in HIRI mice, GSTD treatment was able to enhance *HO-1* mRNA expression levels. Taken together, GSTD may exert an anti-apoptotic role by targeting HO-1 in HIRI mice.

### GSTD alleviated HIRI due to an anti-inflammatory and antioxidant effect

**Figure 5:**
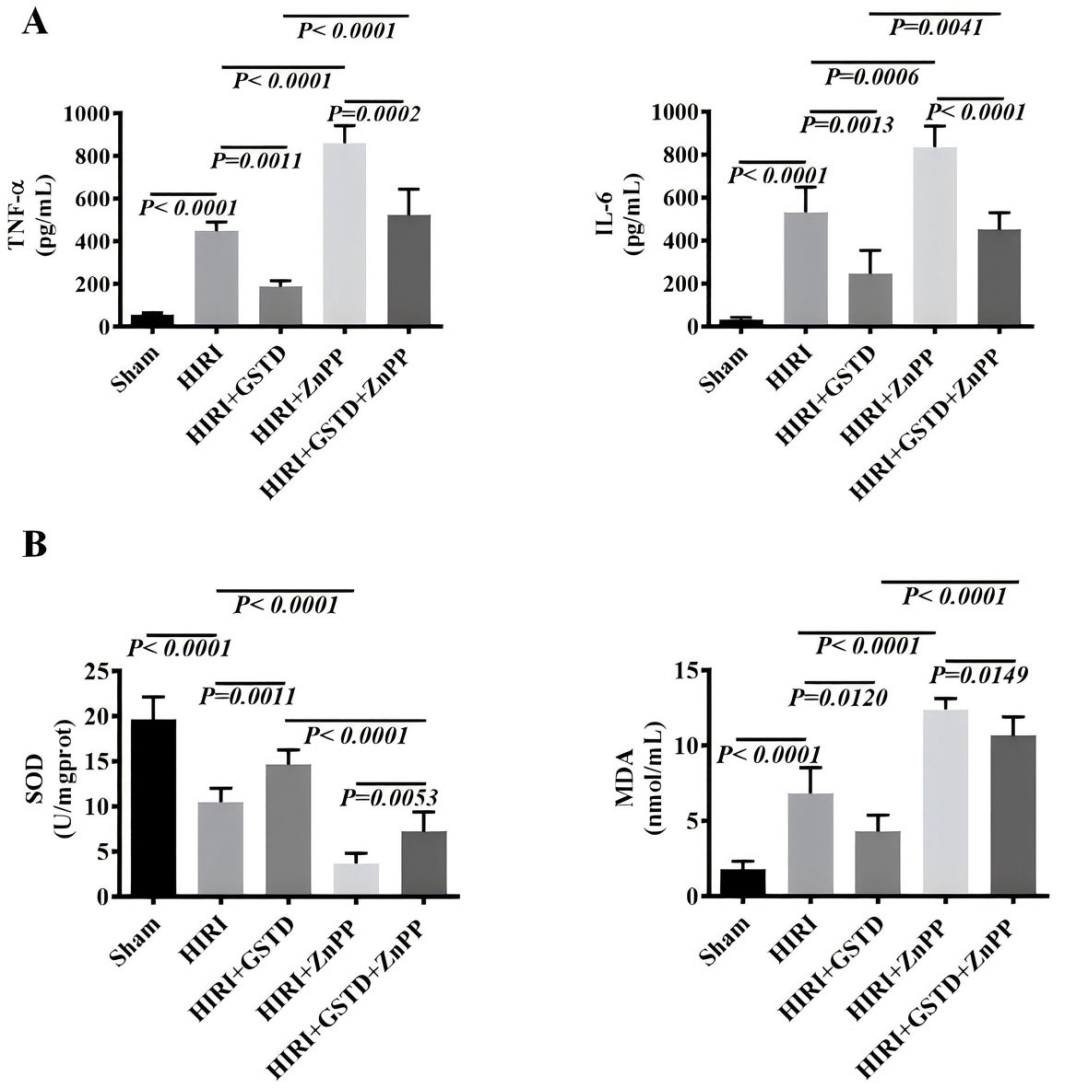
Effect of gastrodin (GSTD) on the level of inflammatory factors tumor necrosis factor (TNF-α) and interleukin (IL)-6 (**A**) and oxidative-stress factors superoxide dismutase (SOD) and malondialdehyde (MDA) (**B**) in the liver of hepatic ischemia reperfusion injury (HIRI) mice model. Data are reported as means and SD (ANOVA). ZnPP: zinc protoporphyrin (HO-1 inhibitor).

In addition to anti-apoptotic effects, GSTD may alleviate HIRI by anti-inflammatory and antioxidant effects (24,25). Therefore, the expression of several proinflammatory and antioxidant factors (TNF-α, IL-6, SOD, and MDA) were measured (Figure 5A and B). Liver TNF-α, IL-6, and MDA of mice in the HIRI group were notably higher than those of animals of the sham group. GSTD decreased liver TNF-α, IL-6, and MDA of HIRI mice since livers of mice in the GSTD+HIRI group showed lower levels of TNF-α, IL-6, and MDA than those compared to the HIRI group. Liver TNF-α, IL-6, and MDA levels were further increased in mice in the HIRI+ZnPP group compared to those of the HIRI group. However, liver levels of TNF-α, IL-6, and MDA in mice in the HIRI+ZnPP+GSTD group were markedly lower than those in the HIRI+ZnPP group. Changes in TNF-α, IL-6, and MDA levels revealed that GSTD decreased these levels in HIRI mice. Liver levels of SOD in mice of the HIRI group were significantly lower than those in the sham group. Mice in the HIRI+GSTD group displayed higher liver SOD levels than those in the HIRI group. Liver SOD levels of mice in the HIRI+GSTD+ZnPP group were also higher than those in the HIRI+ZnPP group. The change in SOD indicated that GSTD induced higher liver SOD levels in HIRI mice.

### Validation of the anti-apoptotic effect of GSTD by targeting HO-1 expression *in vitro*

**Figure 6:**
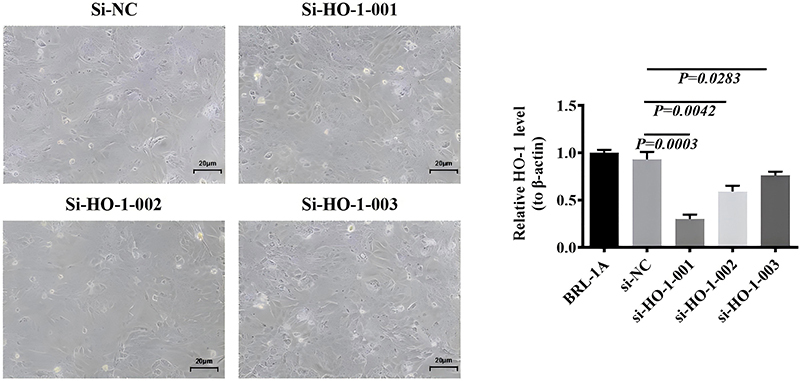
Construction of small interfering heme oxygenase-1 (si-HO-1) cells. Scale bar 20 μm. Data are reported as means and SD (ANOVA).

Three siRNAs (si-HO-1-001, si-HO-1-002, si-HO-1-003) were transfected into BRL-3A cells to inhibit the expression of HO-1, of which si-HO-1-001 was selected to be investigated because of the relatively higher transfection efficiency (Figure 6). GSTD showed no significant cytotoxicity against si-HO-1-001 and BRL-3A cells (IC_50_ >100 µM, data not shown) in preliminary experiments. Concentrations of 10, 50, and 100 µM GSTD were selected to investigate their effect on gene expression.

**Figure 7:**
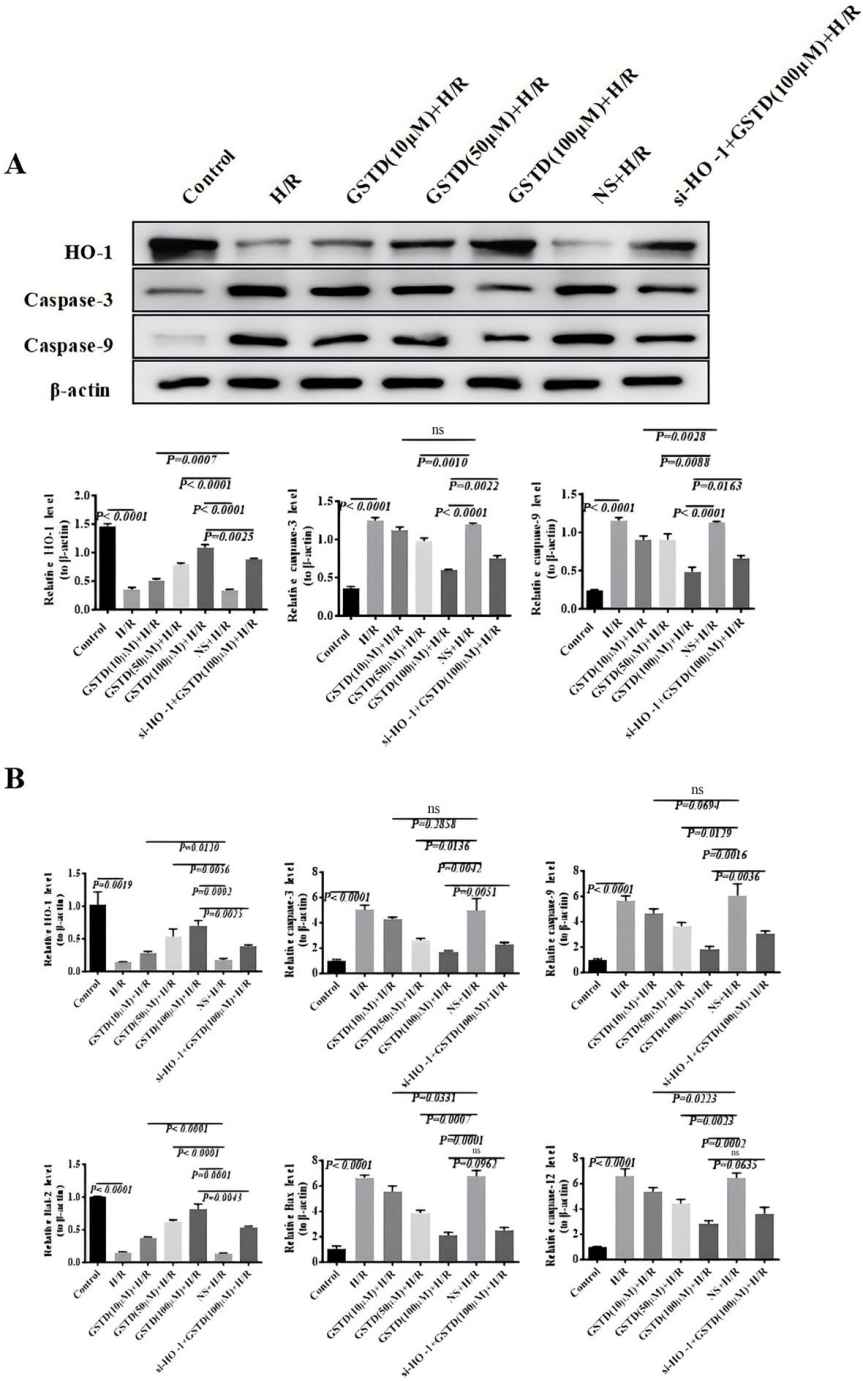
Effect of small interfering heme oxygenase-1 (si-HO-1) on the protein and m-RNA expressions of caspase-3, -9, -12, *Bcl-2*, and *Bax* in gastrodin (GSTD)-treated cells. **A**, Protein expression; **B**, m-RNA expression. Data are reported as means and SD (ANOVA; ns: not significant). H/R: hepatic ischemia/reperfusion; NS: normal saline.

The HO-1 protein expression level in H/R untransfected cells was up-regulated with the addition of GSTD in a dose-dependent manner (Figure 7A). The 100 µM GSTD dose was the most effective concentration to induce HO-1 protein expression in H/R untransfected cells. However, HO-1 protein expression in cells of the H/R+si-HO-1+100 µM GSTD treatment group was notably lower than that in untransfected cells (H/R+100 µM GSTD group). The change of the expression of caspase-3 and -9 proteins was similar, but contrasted with that of HO-1 protein expression. The protein expression of caspase-3 and -9 decreased with an increase in GSTD concentration, which was significantly higher in the H/R+si-HO-1+100 µM GSTD group than in untransfected cells (H/R+100 µM GSTD group). This observation suggested that the down-regulation of HO-1 by RNA interference led to the up-regulation of caspase-3 and -9.

Liver HO-1 mRNA expression levels in mice from the H/R+si-HO-1+100 µM GSTD treatment group were significantly lower than those in mice in the H/R+100 µM GSTD group. The mRNA expression levels of apoptotic genes (caspase-3, -9, -12, and Bax) in untransfected BRL-3A cells negatively correlated with the addition of GSTD (Figure 7B). Moreover, liver mRNA levels of caspase-3 and -9 in mice in the H/R+si-HO-1+100 µM GSTD treatment group were consistently lower than those of mouse livers from the H/R+100 µM GSTD group. Nevertheless, si-HO-1 interference had no significant effect on the expression of caspase-12 and Bax. No significant difference was found between mice in the HR+ si-HO-1+100 µM GSTD and H/R+100 µM GSTD groups.

### Validation of the anti-inflammatory and anti-oxidative effects of GSTD by targeting HO-1 *in vitro*

**Figure 8:**
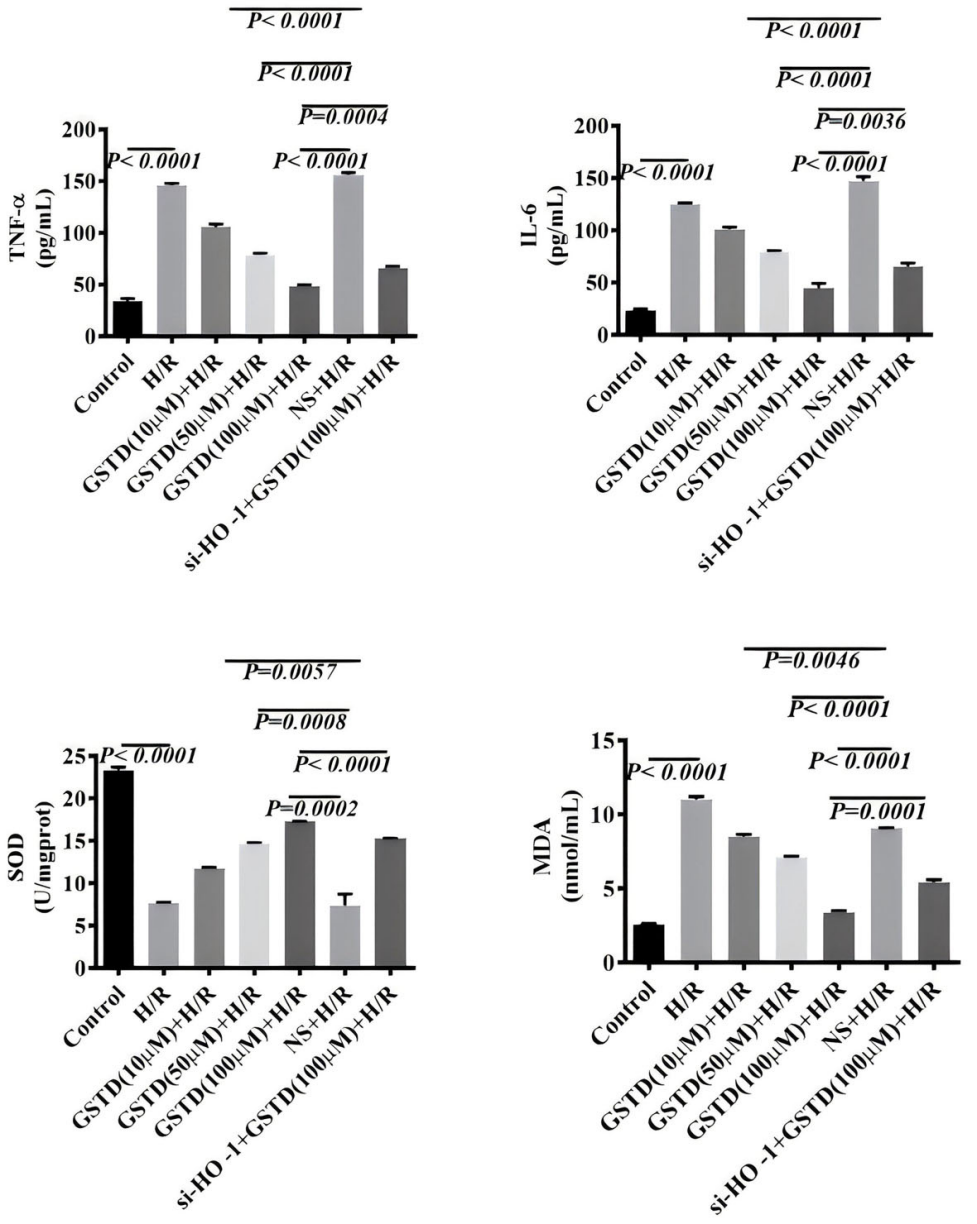
Levels of inflammatory markers tumor necrosis factor (TNF-α) and interleukin (IL)-6 (**A**) and oxidative-stress markers superoxide dismutase (SOD), and malondialdehyde (MDA) (**B**) in gastrodin (GSTD)-treated cells. Data are reported as means and SD (ANOVA). H/R: hepatic ischemia/reperfusion; NS: normal saline.

The protein levels of TNF-α, IL-6, SOD, and MDA were measured in cells by ELISA. We found that the levels of TNF-α, IL-6, and MDA in H/R untransfected BRL-3A cells negatively correlated with GSTD dose (Figure 8). Moreover, liver levels of TNF-α, IL-6, and MDA in mice in the si-HO-1+H/R+100 µM GSTD treatment group were significantly higher than those in the H/R+100 µM GSTD group (Figure 8). The level of SOD also positively correlated with the dose of GSTD. Mice in the si-HO-1+H/R+100 µM GSTD treatment group displayed a lower liver SOD level than those in the H/R+100 µM GSTD group. These observations suggested that HO-1 suppressed oxidation stress and inflammation in H/R BRL-3A cells.

### GSTD bound to HO-1 protein by hydrogen bond

**Figure 9:**
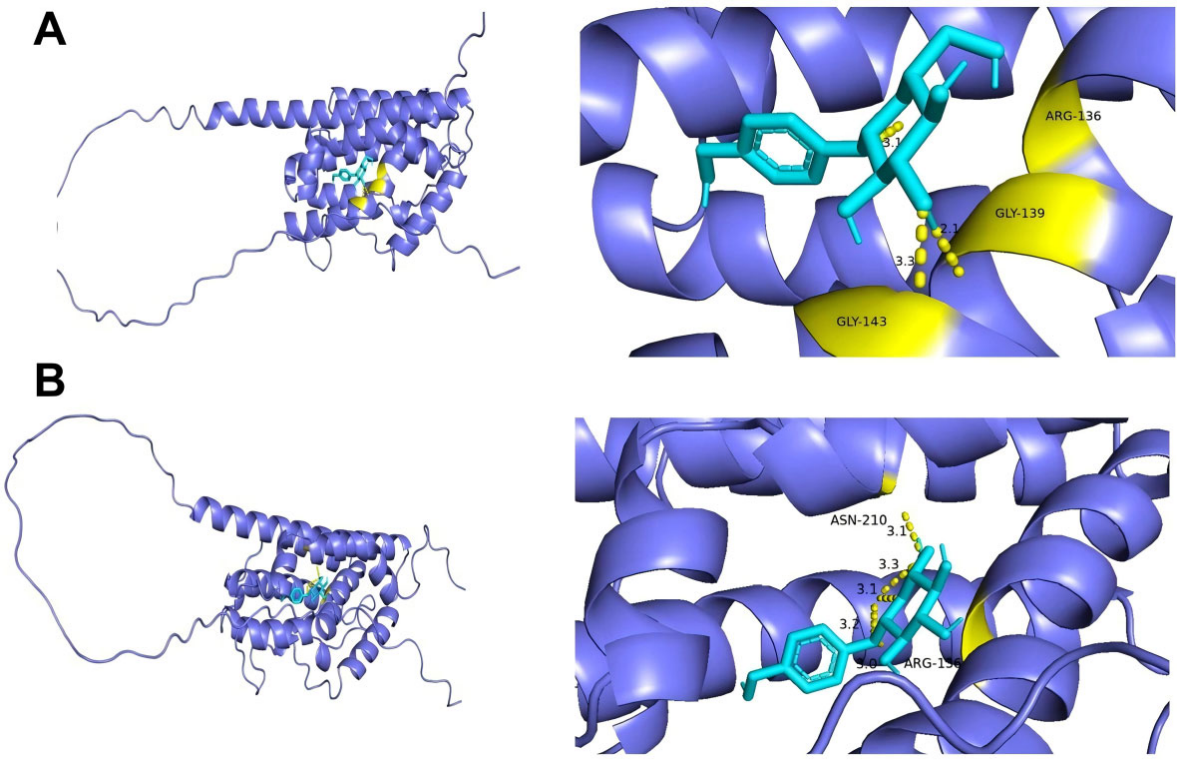
Molecular docking of gastrodin (GSTD) with heme oxygenase-1 (HO-1) protein. **A**, Mouse HO-1 protein; **B**, human HO-1 protein.

As shown in Figure 9, GSTD combined with the mouse HO-1 protein by a hydrogen bond with a binding energy of -7.7 kcal/mol. The binding sites were ARG-136, GLY-139, and GLY-143 in HO-1. GSTD also bound to human HO-1 protein by hydrogen bond with ASN-210 and ARG-136 (binding energy =-7.3 kcal/mol).

## Discussion

HIRI is a complicated pathophysiological event that occurs after the liver undergoes transient ischemia and hypoxia. Damage from HIRI to tissues occurs mainly due to oxidative stress, apoptosis, and inflammation (26-28). GSTD was demonstrated to possess anti-oxidation and anti-inflammatory activity in several prior studies. We investigated the protective role of GSTD in HIRI. The experiments *in vivo* revealed that GSTD was efficient in reducing liver damage in HIRI. This conclusion was drawn from the reduced levels of ALT and glutamic AST, reduced tissue swelling/necrosis and the infiltration of inflammatory cells, and decreased TUNEL staining in the livers of mice in the GSTD+HIRI treatment group compared to those in the HIRI group. The alleviation of HIRI by GSTD was chiefly attributed to anti-apoptotic, anti-inflammatory, and anti-oxidation effects. In accordance with our conclusion, caspase-3 and -9 were down-regulated in mouse livers of the GSTD-treated group. Levels of TNF-α, IL-6, and MDA were reduced in the GSTD-treated group.

In our previous study, *in vivo* experiments demonstrated that the HO-1 mRNA level was up-regulated in a HIRI mouse model with GSTD treatment, suggesting that GSTD may protect HIRI by regulating HO-1 (21). Therefore, we further revealed a role for GSTD in regulating HO-1 in the alleviation of HIRI. *In vivo* experiments revealed that both HO-1 protein and mRNA levels were down-regulated in HIRI mice. GSTD action was antagonistic to the down-regulation of HO-1 mRNA and protein in HIRI mice. HO-1 was induced by GSTD, thus partially compensating for the down-regulation of HO-1 in the HIRI mice model. The use of ZnPP, a specific inhibitor of HO-1, further reduced the HO-1 expression in HIRI mice. However, co-administration of GSTD and ZnPP displayed a higher level of HO-1 than that without GSTD administration (ZnPP +HIRI group). Thus, it was safely concluded that GSTD may up-regulate HO-1 in HIRI mice. The anti-apoptotic role of HO-1 in liver and kidney cells and in cardiac myocytes has been well documented (29-32).

In the present study, levels of apoptosis-associated proteins (caspase-3 and -9) and mRNA (caspase-3, -9, -12, and *Bax*) were down-regulated with the enhancement of HO-1 expression in response to GSTD. The mRNA level of the anti-apoptotic protein, Bcl-2, was up-regulated with the enhancement of HO-1 expression due to GSTD. The above observation suggested an anti-apoptotic role for GSTD in regulating HO-1 expression, which may protect the liver from pathological injury during the ischemic stage in HIRI.

The anti-inflammatory and anti-oxidation effect of HO-1 has also been reported in several previous investigations (33-35). The levels of TNF-α, IL-6, and MDA were reduced when HO-1 expression was enhanced in livers of mice in the GSTD treatment group. This illustrated that the anti-inflammatory and anti-oxidation effect of GSTD may be also attributed to HO-1 expression, which would reduce inflammation and oxidative injury in tissues during the reperfusion stage in HIRI. The other straightforward evidence of GSTD regulating HO-1 comes from the results of HO-1 mRNA interference. The down-regulation of HO-1 by RNA interference enhanced caspase-3 and -9 mRNA and protein expression in si-HO-1 cells compared to un-transfected cells in the H/R model. Higher levels of TNF-α, IL-6, and MDA were also observed in si-HO-1 cells compared to un-transfected cells in the H/R model. The down-regulation of HO-1 by si-RNA interference weakened the anti-apoptotic, anti-inflammatory, and anti-oxidative protective role of GSTD in H/R cells.

In an analysis of molecular docking, we showed, for the first time, that GSTD was able to combine with mice and human HO-1 proteins through hydrogen bonds with low binding energies of -7.7 and -7.3 kcal/mol, respectively. This provided evidence that GSTD regulated HO-1 from the aspect of a structural relationship of drug molecule and drug target. Overall, the alleviation of HIRI by GSTD was chiefly attributed to anti-apoptotic, anti-inflammatory, and anti-oxidation action by regulating HO-1 expression.

Although GSTD was demonstrated to play a protective role in HIRI by regulating HO-1 *in vivo*, a limitation of this study was that confirmation of GSTD regulating HO-1 by RNA interference was not achieved in the HIRI model compared to the HR model *in vitro*. The main reason was a technical barrier for the establishment of a HIRI cell model *in vitro* (36,37). An apparent deficiency of our investigation is that evidence for GSTD regulation of HO-1 to protect HIRI *in vitro* was not clear. A prospective study should evaluate the pharmacological effect of GSTD in HO-1 transgenic mice under HIRI conditions. In addition, HO-1 is a common protective gene in many cells and tissues under stress conditions, and is involved in a complicated regulatory system (36,38). The revelation of a HO-1 cored net system regulated by GSTD in alleviating HIRI injury also needs extensive and elaborative investigations in the future. Another limitation of this study was that the GSTD regulation of HO-1 to alleviate HIRI was confirmed in an *in vivo* mouse model. The real effect of GSTD on human HIRI remains unclear and further clinical research is needed.

GSTD regulates HO-1 expression to play a protective role in HIRI by anti-apoptotic, anti-inflammatory, and antioxidant effects. The application of GSTD in alleviating HIRI in liver surgery looks promising, as it has a few advantages. First of all, GSTD has been used as a drug to prevent cardiovascular and cerebrovascular diseases in China, so the clinical safety of GSTD is well known. Second, GSTD is a rich natural compound in *Gastrodia elata.* The preparation of GSTD is not complicated, which ensures a commercial supply of GSTD. The use of GSTD may be an alternative strategy to alleviate the effect of HIRI in the future if clinical research with GSTD is successful.
